# Investigation of activation-induced markers (AIM) in porcine T cells by flow cytometry

**DOI:** 10.3389/fvets.2024.1390486

**Published:** 2024-05-29

**Authors:** Madison Moorton, Priscilla Y. L. Tng, Ryo Inoue, Christopher L. Netherton, Wilhelm Gerner, Selma Schmidt

**Affiliations:** ^1^The Pirbright Institute, Woking, United Kingdom; ^2^School of Biological Sciences, University of Reading, Whiteknights, Reading, United Kingdom; ^3^Laboratory of Animal Science, Setsunan University, Osaka, Japan

**Keywords:** activation-induced markers, CD69, CD40L, CD25, ICOS, SEB, African swine fever virus, pig

## Abstract

Activation-induced markers (AIMs) are frequently analyzed to identify re-activated human memory T cells. However, in pigs the analysis of AIMs is still not very common. Based on available antibodies, we designed a multi-color flow cytometry panel comprising pig-specific or cross-reactive antibodies against CD25, CD69, CD40L (CD154), and ICOS (CD278) combined with lineage/surface markers against CD3, CD4, and CD8α. In addition, we included an antibody against tumor necrosis factor alpha (TNF-α), to study the correlation of AIM expression with the production of this abundant T cell cytokine. The panel was tested on peripheral blood mononuclear cells (PBMCs) stimulated with phorbol 12-myristate 13-acetate (PMA)/ionomycin, Staphylococcus enterotoxin B (SEB) or PBMCs from African swine fever virus (ASFV) convalescent pigs, restimulated with homologous virus. PMA/ionomycin resulted in a massive increase of CD25/CD69 co-expressing T cells of which only a subset produced TNF-α, whereas CD40L expression was largely associated with TNF-α production. SEB stimulation triggered substantially less AIM expression than PMA/ionomycin but also here CD25/CD69 expressing T cells were identified which did not produce TNF-α. In addition, CD40L-single positive and CD25^+^CD69^+^CD40L^+^TNF-α^−^ T cells were identified. In ASFV restimulated T cells TNF-α production was associated with a substantial proportion of AIM expressing T cells but also here ASFV-reactive CD25^+^CD69^+^TNF-α^−^ T cells were identified. Within CD8α^+^ CD4 T cells, several CD25/CD40L/CD69/ICOS defined phenotypes expanded significantly after ASFV restimulation. Hence, the combination of AIMs tested will allow the identification of primed T cells beyond the commonly used cytokine panels, improving capabilities to identify the full breadth of antigen-specific T cells in pigs.

## 1 Introduction

The identification of effector cells is a key element to understanding the contribution of T cells in immune responses. Detection of molecules or markers associated with different effector functions by polychromatic or high-dimensional flow cytometry (FCM) is one approach to detect such cells. For example, analyzing one or several cytokines can aid in the identification of Th1, Th2, Th17, or Treg cells (1). However, depending on the cytokine, release from T cells varies over time (2). In addition, for intracellular cytokine staining by FCM, the Golgi apparatus needs to be inhibited for prevention of cytokine release from the cell (3). This makes it difficult to identify all effector T cells by intracellular cytokine staining, even if several cytokines are investigated in combination.

Due to these limitations, the investigation of Activation-induced markers (AIMs), often in combination with one or several cytokines has become the preferred method in studies on human T cell responses (4–9). However, in livestock species, the use of AIMs for identification of activated T cells is limited, mainly due to restricted availability of antibodies to detect markers used for human T cells, like OX40 (CD134), 41BB (CD137), CD200 or PD-L1 (CD274). However, for other molecules qualifying as AIMs, like CD25, CD69, CD40L (CD154) or inducible T-cell co-stimulator (ICOS, CD278), either species-specific or cross-reactive antibodies are available for porcine T cells (10–14).

CD25 is the α-chain of the IL-2 receptor and is expressed on activated CD4 T cells but also regulatory T cells (Tregs); this was also demonstrated for porcine T cells (15, 16). Due to the ubiquitous expression on Tregs, CD25 expression is analyzed in combination with other AIMs in the identification of activated T cells (8, 9). CD69 is a type II C lectin receptor. For pigs, a mAb only recently became available (12) and it was shown that subsets of CD8 T cells in lymph nodes and lung express CD69 under steady state conditions (17). In human T cells, CD69 expression already peaks about 18 h after stimulation (18). The marker is frequently analyzed in combination with CD25 or other AIMs for the identification of activated CD8 T cells (8). CD40L belongs to the tumor necrosis factor (TNF) superfamily and is typically expressed by CD4 T cells following T cell receptor (TCR) stimulation but also CD8 T cells (19–21). For pigs, it has been shown that expression of CD40L correlates in CD4 T cells with production of interferon (IFN)-γ and TNF-α both after *Staphylococcus* enterotoxin B (SEB) stimulation and restimulation with *Candida albicans, Ascaris suum* and *Streptococcus suis* antigens *in vitro* (11). ICOS has been used less frequently as an AIM. For human CD4 T cells it was investigated to identify activated circulating T follicular helper cells (Tfh) (22, 23) but also total activated CD4 T cells (23). In pigs, a cross-reactive antibody to ICOS has been used to investigate invariant natural killer T (iNKT) cells in different lymphoid and non-lymphoid organs (14). In addition, ICOS expression was studied in the context of lymph node and blood-derived porcine Tfh cells (13).

Based on this, we tested combinations of antibodies against CD25, CD69 and CD40L to identify activated CD4 and CD8 T cells in pigs, using polyclonal (phorbol 12-myristate 13-acetate [PMA]/ionomycin) and oligoclonal (SEB) stimulation. The three AIMs were also combined with ICOS and tested in the context of antigen-specific restimulation, using peripheral blood mononuclear cells (PBMCs) from African swine fever virus (ASFV) convalescent pigs. Comparing expression patterns of these AIMs against TNF-α, one of the most widely expressed porcine cytokines in the context of T cell activation (24), we show that combinations of CD25 and CD69 have the capacity to identify additional antigen-specific T cells, while CD40L was largely associated with TNF-α production.

## 2 Materials and methods

### 2.1 ASFV for *in vitro* cultivation

The moderately virulent Estonia 2014 ASFV strain [spleen homogenate kindly provided by Sandra Blome from Friedrich-Loeffler-Institute, Greifswald-Insel Riems, Germany (25)] was used for *in vitro* restimulation experiments and cultured and titrated using end point dilution on bone-marrow-derived macrophages as detailed in a previous study (26). In brief, bone marrow cells were extracted from femurs of 4–6 week old outbred pigs and cultured in EBSS (Sigma) supplemented with 4 mM HEPES, 10% heat-inactivated pig serum (BioSera), and 100 I.U./mL penicillin with 100 ug/mL streptomycin (Gibco) for 3 days prior to ASFV infection to allow the differentiation of bone-marrow-derived macrophages. Mock inoculum was prepared from the same stock of uninfected cells. Virus was collected from the cells 5 days post-infection and titrated using the Spearman-Karber method of end-point dilution, wherein 50% of infected bone-marrow-derived macrophage cultures exhibited haemadsorption caused by ASFV.

### 2.2 Cell isolation and animals used in the study

Peripheral blood mononuclear cells (PBMCs) were isolated from heparinized blood by density gradient centrifugation (Histopaque-1077 Hybri-Max, density 1.077 g/mL, Sigma-Aldrich, Merck KGaA, Darmstadt, Germany). Blood for *in vitro* assays with PMA/ionomycin and SEB was obtained postmortem from six outbred pigs, aged 4 to 6 weeks. The animal experiment involving ASFV was conducted under the Home Office Animals (Scientific Procedures) Act (1986) (ASPA) and was approval by the Animal Welfare and Ethical Review Board (AWERB) at The Pirbright Institute. The animals were housed in accordance with the Code of Practice for the Housing and Care of Animals Bred, Supplied, or Used for Scientific Purposes. Throughout the study, appropriate bedding and species-specific enrichment measures were implemented to uphold high standards of welfare. All procedures were performed by trained and competent Personal License holders under the authority of Project License PP8739708. In detail, four female (animals AY95, AY97, AY98 and AY99) and one male (animal AY94) 12-week-old Babraham pigs were bred at the Centre for Dairy Research, University of Reading, Whiteknights, United Kingdom. The pigs were acclimatized for a week before oronasal challenge with 1,145 HAD_50_ moderately virulent ASFV strain Estonia 2014 in spleen homogenate. The challenge dose of ASFV was confirmed by back titration on bone-marrow-derived macrophages. Clinical signs and macroscopic lesion at post-mortem were assessed as previously described (27, 28). All animals survived the viral challenge. Heparinized blood samples were obtained from the animals 21 days post viral challenge. Following careful monitoring all pigs were euthanized using an overdose of anesthetic after reaching scientific or humane endpoints.

### 2.3 *In vitro* cultivation and FCM staining

Round-bottom 96-well microtiter plates (Nunc MicroWell Plates, Thermo Fisher Scientific, Waltham, MA, United States) were seeded with 5 × 10^5^ thawed (PMA and SEB) or freshly isolated PBMCs (PMA and ASFV Estonia 2014) in a final volume of 200 μL/well in RPMI-1640 Medium (Sigma-Aldrich) supplemented with 100 U/mL penicillin, 0.1 mg/mL streptomycin (Gibco, Thermo Fisher Scientific) and 10% heat-inactivated fetal bovine serum (FBS, Life Science Production, Life Science Group, Sandy, United Kingdom). Cells were incubated with PMA (5 ng/mL) and ionomycin (500 ng/mL), SEB (500 ng/mL, all Sigma-Aldrich), or ASFV Estonia 2014 at a multiplicity of infection of 0.5 for 18 h. Cells incubated in cell culture medium only or incubated with mock inoculum served as negative controls. Brefeldin A (BD GolgiPlug™, BD Biosciences, San Jose, CA, United States) was added to the cultures for the final 6 h of cultivation at a concentration of 1 μg/mL. After 18 h, cells were harvested and re-suspended in staining buffer containing PBS with 3% FBS (Life Science Production). Cells were surface-stained with primary monoclonal antibodies (mAbs) directed against CD3 (PerCP-Cy5.5-conjugated, mouse IgG2a anti-pig, clone: BB23-8E6-8C8, BD Biosciences), CD4 (FITC-conjugated, mouse IgG1 anti-pig, clone: b38c6c7, Bio-Rad Laboratories Ltd., Hercules, CA, United States), CD8α (biotinylated, mouse IgG2a anti-pig, clone: 76–2-11, Southern Biotech, Birmingham, AL, United States), CD25 (AlexaFluor 647-conjugated, mouse IgG1 anti-pig, clone: K231.3B2, Bio-Rad) and CD69 [unconjugated, mouse IgG2b anti-pig, clone: 01-14-22-51 (12)]. Cells derived from ASFV Estonia 2014-stimulated cultures were additionally stained with a primary monoclonal antibody against ICOS (CD278, BV605-conjugated, anti-human/mouse/rat, hamster IgG, clone: C398.4A, BioLegend, San Diego, CA, United States). Streptavidin-BV421 (BioLegend) and goat anti-mouse-IgG2b-PE (Tonbo, Cytek Biosciences, Fremont, CA, United States) were added in a secondary staining step, to label CD8α and CD69, respectively. Dead cells were identified using Fixable Viability Dye eFluor780 (Thermo Fisher Scientific) after surface staining according to the manufacturer’s instructions. BD Cytofix/Cytoperm™ Fixation/Permeabilization Kit (BD Biosciences) was used following the manufacturer’s instructions by adding 100 μL per well of Fixation/Permeabilization solution to resuspended cells for 20 min at 4°C, followed by two washes with Perm/Wash Buffer (BD Biosciences). Intracellular staining was performed using the following mAbs: TNF-α-BV711 (mouse IgG1 anti-human, clone: Mab11, BioLegend) and CD40L-PE-Vio770 (CD154, anti-human, recombinant human IgG1, clone: REA238, Miltenyi Biotec, Bergisch Gladbach, Germany). Staining steps were carried out in 96-well round bottom plates at 4°C for 20 min with the exception of the intracellular incubation step which lasted 30 min. Following intracellular incubation, cells were washed twice and stored at 4°C overnight in 50 μL Perm/Wash Buffer (BD Biosciences). Samples were acquired the following morning on a Cytek Aurora Spectral Cytometer (Cytek Biosciences), equipped with 5 lasers (UV 355 nm, violet 405 nm, blue 488 nm, yellow-green 561 nm, red 640 nm) and 64 fluorescence detection channels UV: 16, violet: 16, blue: 14, yellow-green: 10, red: 8. Spectral unmixing was performed using SpectroFlo software (version 3.2.1, Cytek Biosciences) following the acquisition of single-stained reference samples. Autofluorescence signatures based on unstained controls were extracted from the samples. Data of a minimum of 1 × 10^5^ live lymphocytes per sample were recorded and analyzed on FlowJo Software for Windows (Version 10.9.0, BD Biosciences). Boolean “AND” combination gates were used to define co-expression of CD25^+^, CD40L^+^, CD69^+^, TNF-α^+^ and ICOS^+^ in T cells. Boolean gating resulted in 16 possible phenotypes for combination gates including CD25, CD40L, CD69 and TNF-α as well as 32 possible phenotypes for combination gates including CD25, CD40L, CD69, TNF-α and ICOS.

### 2.4 Statistical analysis

Statistical analysis was performed using GraphPad Prism 9 (GraphPad Software, Dotmatics, Boston, MA, United States). Data sets were analyzed for normality. A paired t-test was used to test for statistical differences between groups where the data was normally distributed. A Wilcoxon matched-pairs signed rank test was used for data sets that were not normally distributed. A *p-*value of ≤0.05 was considered statistically significant. t-SNE plots were generated in R (version 4.2.3). t-SNE algorithm was run on live CD3^+^ T cells using the parameters CD4, CD8α, CD25, CD40L, CD69 and TNF-α with samples of five pigs per stimulation condition (Medium, SEB, PMA). The script used was developed by the group of Adrian Liston (29) and is available on GitHub at https://github.com/AdrianListon/Cross-Entropy-test.

## 3 Results

### 3.1 PMA/ionomycin leads to strong upregulation of AIMs in pigs

To investigate the suitability of CD25, CD69 and CD40L as AIMs in pigs, we started by stimulating PBMCs from healthy animals with PMA/ionomycin. PMA works through the activation of protein kinase C while ionomycin triggers calcium release, thus bypassing TCR engagement. In combination, PMA/ionomycin is a very potent polyclonal stimulant for T cell activation. Cells cultured in medium only served as negative controls. To focus on T cells, CD3^+^ cells were gated within live lymphocytes (Supplementary Figure 1).

**Figure 1:**
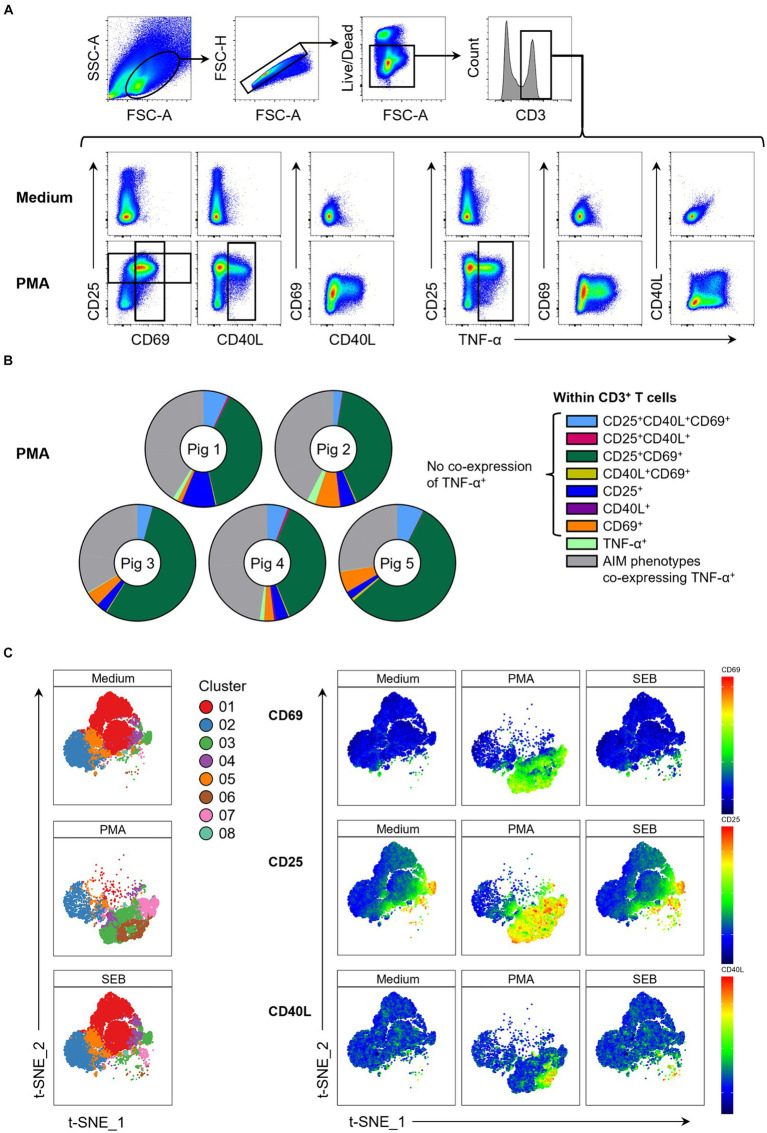
Expression of AIMs in blood-derived CD3^+^ T cells after stimulation with PMA/ionomycin. **(A)** Representative FCM plots depicting expression of CD25, CD69, CD40L and TNF-α in CD3^+^ T cells when PBMC samples were unstimulated (Medium, top row) or stimulated with PMA/ionomycin (PMA, bottom row) for 18 h. Surface staining was performed for antibodies against CD3, CD4, CD8α, CD25 and CD69. Intracellular staining was performed for CD40L and TNF-α. Gates shown are representative of gating for total CD25^+^, total CD69^+^, total CD40L^+^ and total TNF-α^+^ T cells applied to PMA-stimulated samples and used in Boolean gating to create doughnut charts. **(B)** Doughnut charts of AIM phenotypes in PMA-stimulated samples generated by Boolean gating. Each doughnut represents the PBMC sample of one pig. Different phenotypes are indicated by different colors with all AIM phenotypes co-expressing TNF-α summarized in gray. CD25^−^CD40L^−^CD69^−^TNF-α^−^ T cells are not shown. **(C)** Live CD3^+^ T cells from unstimulated (Medium), SEB-stimulated and PMA-stimulated cultures were clustered using the t-SNE algorithm with generated clusters shown in a colored overlay (left side). Relative expression levels of CD69, CD25 and CD40L within clusters (right side) are colored from high (red) to low (blue).

The investigated AIMs CD25, CD69 and CD40L were strongly upregulated in PMA-stimulated samples compared to the medium control (Figure 1A). In addition, a large proportion of AIM^+^ cells showed co-expression of TNF-α. To elucidate individual T cell phenotypes further, CD3^+^ T cells were further gated for CD25^+^, CD69^+^, CD40L^+^ and TNF-α^+^ cells, resulting in 16 possible phenotypes identified by Boolean gating (Supplementary Figure 2). PMA/ionomycin stimulation resulted in substantial upregulation of the AIMs under investigation, with 85 to 95% of T cells showing an AIM^+^ phenotype (Supplementary Table 1). Most prominent phenotypes induced by PMA/ionomycin were CD25^+^CD69^+^ (green), CD25^+^CD40L^+^CD69^+^ (light blue), CD69 single^+^ (orange) and CD25 single^+^ (dark blue) T cells (Figure 1B). AIM^+^ T cell phenotypes co-expressing TNF-α (collectively highlighted in gray) only constituted between 26% and 43% of total T cells (Supplementary Table 1), showing that analysis of AIMs identifies additional PMA-responding T cells.

We further used a t-SNE algorithm to explore T cell phenotypes induced by PMA stimulation and visualize these in contrast to medium control samples. t-SNE plots revealed three clusters that were uniquely present in PMA-stimulated samples (Figure 1C; Supplementary Figure 3): cluster 3 (CD25^high^CD69^+^partiallyCD40L^+^), cluster 6 (CD25^high^CD69^+^CD40L^+^TNF-α^+^) and cluster 7 (CD25^high^CD69^+^). While CD69 and CD40L expression was almost entirely restricted to PMA-stimulated samples, variable level CD25 expression was also present in medium controls. Most of these cells co-expressed CD4 (Supplementary Figure 3). Cluster 3 in medium consisted almost exclusively of CD25^high^ CD4^+^ T cells, most likely representing Tregs. Thus, PMA/ionomycin stimulation of porcine PBMCs strongly upregulated expression of CD25, CD69 and CD40L, confirming these as suitable AIMs in the pig.

### 3.2 AIMs are predominant in CD4^+^CD8α^+^ T cells after SEB stimulation

**Figure 2:**
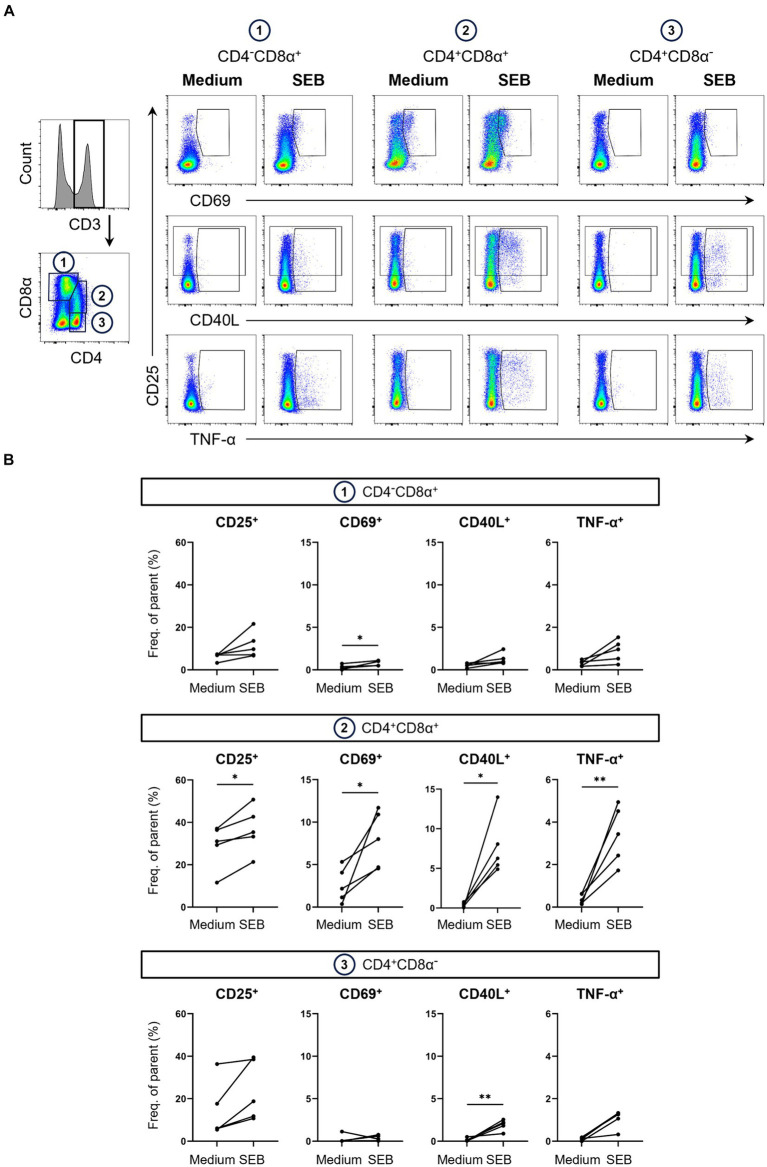
Expression of AIMs in blood-derived CD4/CD8α-defined T cell subsets after stimulation with SEB. **(A)** Representative FCM plots depicting expression of CD25, CD69, CD40L and TNF-α in CD4^−^CD8α^+^, CD4^+^CD8α^+^ and CD4^+^CD8α^−^ T cells. PBMC samples were unstimulated (Medium, left columns) or stimulated with SEB (SEB, right columns) for 18 h. Cells were pre-gated in the following order: live, single and CD3^+^. Gates shown indicate gating applied to calculate frequencies of AIM^+^ and TNF-α^+^ cells. **(B)** Frequencies of CD25^+^, CD69^+^, CD40L^+^ and TNF-α^+^ cells within CD4/CD8α-defined T cell subsets in unstimulated (Medium) and SEB-stimulated samples. Each dot represents data from one animal (*n* = 5). Asterisks indicate significant differences between groups (^*^p ≤ 0.05, ^**^p ≤ 0.01).

Next, we investigated AIM expression after stimulation of PBMCs with SEB. SEB is a superantigen that binds to the α-chain of MHC class II molecules on antigen-presenting cells and specific Vβ chains of the TCR which leads to activation of T cells and cytokine release (30, 31). While still non-specific, SEB activates TCR signaling via similar pathways to when T cells are activated by their cognate MHC peptides (32, 33). t-SNE analysis showed that overall AIM expression levels within total T cells after SEB stimulation were much lower compared to PMA stimulation, however more CD25^+^, CD40L^+^ and CD69^+^ T cells were visible in clusters 6 and 7 compared to medium controls (Figure 1C). As SEB is known to predominantly activate CD4^+^ T cells (34), CD3^+^ T cells were further sub-gated into three populations based on their expression of CD4 and CD8α: CD4^−^CD8α^+^, CD4^+^CD8α^+^ and CD4^+^CD8α^−^ T cells (Figure 2A).

Analysis of AIMs within these subsets revealed that SEB-stimulation led to expression of all AIMs as well as TNF-α production in all three T cell subsets. Frequencies of AIM^+^ T cells within the CD4^−^CD8α^+^ T cell subset, which mostly contains CD8 T cells, were higher in SEB-stimulated samples compared to medium controls (Figure 2B). Due to high animal-to-animal variation, however, only CD69^+^ CD4^−^CD8α^+^ T cells reached a significant difference. CD8α^+^ CD4^+^ T cells are a special subset in the pig with high percentages in blood increasing with age and consisting mainly of activated and/ or memory T cells (35, 36). Among the investigated T cell subsets, highest overall frequencies of AIM-expressing and TNF-α producing cells were observed within CD4^+^CD8α^+^ T cells with all markers expressed in significantly higher frequencies in SEB-stimulated samples than in medium controls. SEB also induced AIM upregulation in the CD4^+^CD8α^−^ T cell subset, largely formed by naïve CD4^+^ T cells (35), with the exception of one pig that showed a decrease in CD69 expression after stimulation.

### 3.3 ASFV induces expression of multiple AIMs

While the *in vitro* stimulation experiments using PMA/ionomycin and SEB confirmed the potential of an AIM assay in the pig, the ultimate goal was to utilize the AIMs for the detection of antigen-specific T cells after immunization or infection. To that end, we employed ASFV-primed cells from five pigs that had undergone a challenge infection with the moderately virulent ASFV strain Estonia 2014. Fresh blood samples were collected 21 days post viral challenge and isolated PBMCs were subjected to 18 h *in vitro* restimulation with ASFV Estonia 2014. Cells incubated with mock inoculum served to determine background expression.

**Figure 3:**
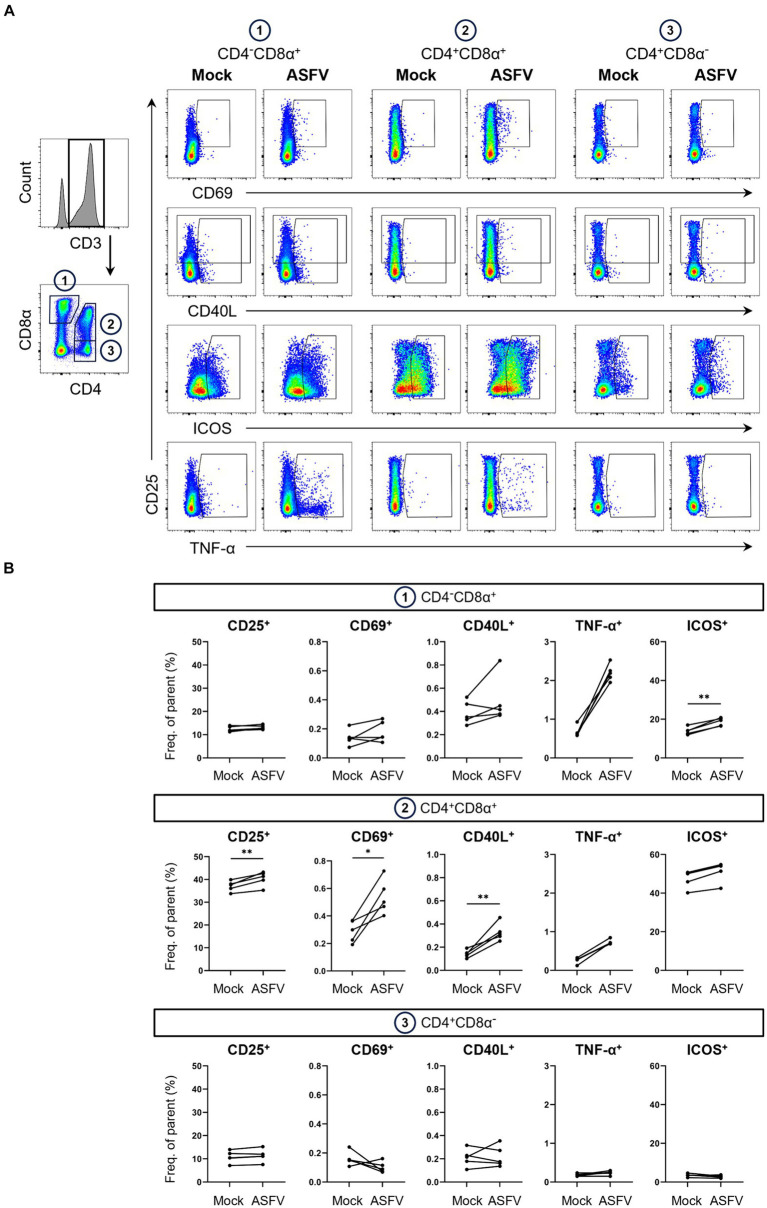
Expression of AIMs in blood-derived CD4/CD8α-defined T cell subsets after stimulation with ASFV Estonia 2014. **(A)** Representative FCM plots depicting expression of CD25, CD69, CD40L, ICOS and TNF-α in CD4^−^CD8α^+^, CD4^+^CD8α^+^ and CD4^+^CD8α^−^ T cells. PBMC samples were incubated with mock inoculum (Mock, left columns) or ASFV Estonia 2014 (ASFV, right columns) for 18 h. Cells were pre-gated in the following order: live, single and CD3^+^. Gates shown indicate gating applied to calculate frequencies of AIM^+^ and TNF-α^+^ cells. Dot size was enlarged to improve visibility of AIM^+^ and TNF-α^+^ cells. **(B)** Frequencies of CD25^+^, CD69^+^, CD40L^+^, ICOS^+^ and TNF-α^+^ cells within CD4/CD8α-defined T cell subsets in mock-inoculated (Mock) and ASFV Estonia 2014 (ASFV)-stimulated samples. Each dot represents data from one animal (n = 5). Asterisks indicate significant differences between groups (^*^p ≤ 0.05, ^**^p ≤ 0.01).

Identical to SEB-stimulated samples, ASFV Estonia-stimulated samples were gated on live CD3^+^ T cells (Supplementary Figure 1, bottom) which were further divided into three CD4/CD8α-defined T cell subsets (Figure 3A). In addition to the analysis of CD25, CD69 and CD40L, ICOS was included as a further potential AIM. Although not traditionally used in human AIM studies, ICOS is known to be expressed at low levels on naïve T cells and upregulated following T cell activation in both CD4 and CD8 T cells (37–39). As predicted when using an antigen-specific stimulation approach, overall levels of AIM expression were lower than those observed with PMA/ionomycin or SEB stimulation (Figure 3B).

Interestingly, highest frequencies of TNF-α in response to ASFV Estonia stimulation were found in CD4^−^CD8α^+^ T cells. Significance was not reached for this phenotype since data from the mock control was not normally distributed. Of note, we observed increases in ICOS^+^ and CD40L^+^ expressing cells in CD4^−^CD8α^+^ T cells, with ICOS even reaching significance, although both molecules are more associated with CD4 T cells. While overall percentages of CD40L expressing cells were low, ASFV Estonia stimulation induced significant CD40L upregulation above mock controls in CD4^+^CD8α^+^ T cells of all pigs. In line with results for SEB, increased frequencies of CD25, CD69 and ICOS expressing cells after ASFV Estonia stimulation were detected in the CD4^+^CD8α^+^ T cell subset. Consistent with the notion that CD4^+^CD8α^−^ cells represent naïve CD4 T cells, no consistent increase of AIM expressing T cells was found in this subset following ASFV stimulation. Similarly, an increase in TNF-α producing cells was found in CD4^+^CD8α^+^ T cells (though not significant) but not in the CD4^+^CD8α^−^ subpopulation. Therefore, ASFV stimulation induced the expression of multiple AIMs, most prominently in the CD4^+^CD8α^+^ T cell subset.

### 3.4 CD25^+^CD69^+^ is a prominent AIM T cell phenotype for SEB and ASFV stimulation

To compare AIM^+^ T cell phenotypes elicited by SEB with the antigen-specific stimulation of ASFV-primed cells, the 16 phenotypes defined by expression of CD25^+^, CD69^+^, CD40L^+^ and TNF-α^+^ cells were analyzed by Boolean gating within CD4^−^CD8α^+^, CD4^+^CD8α^+^ and CD4^+^CD8α^−^ T cells. For both stimulations, CD25 single^+^ T cells strongly dominated in all three T cell subsets (Supplementary Figures 4A,B). As the CD25 single^+^ T cell subset was in its majority likely composed of Tregs, we decided to exclude these cells from further analysis.

**Figure 4:**
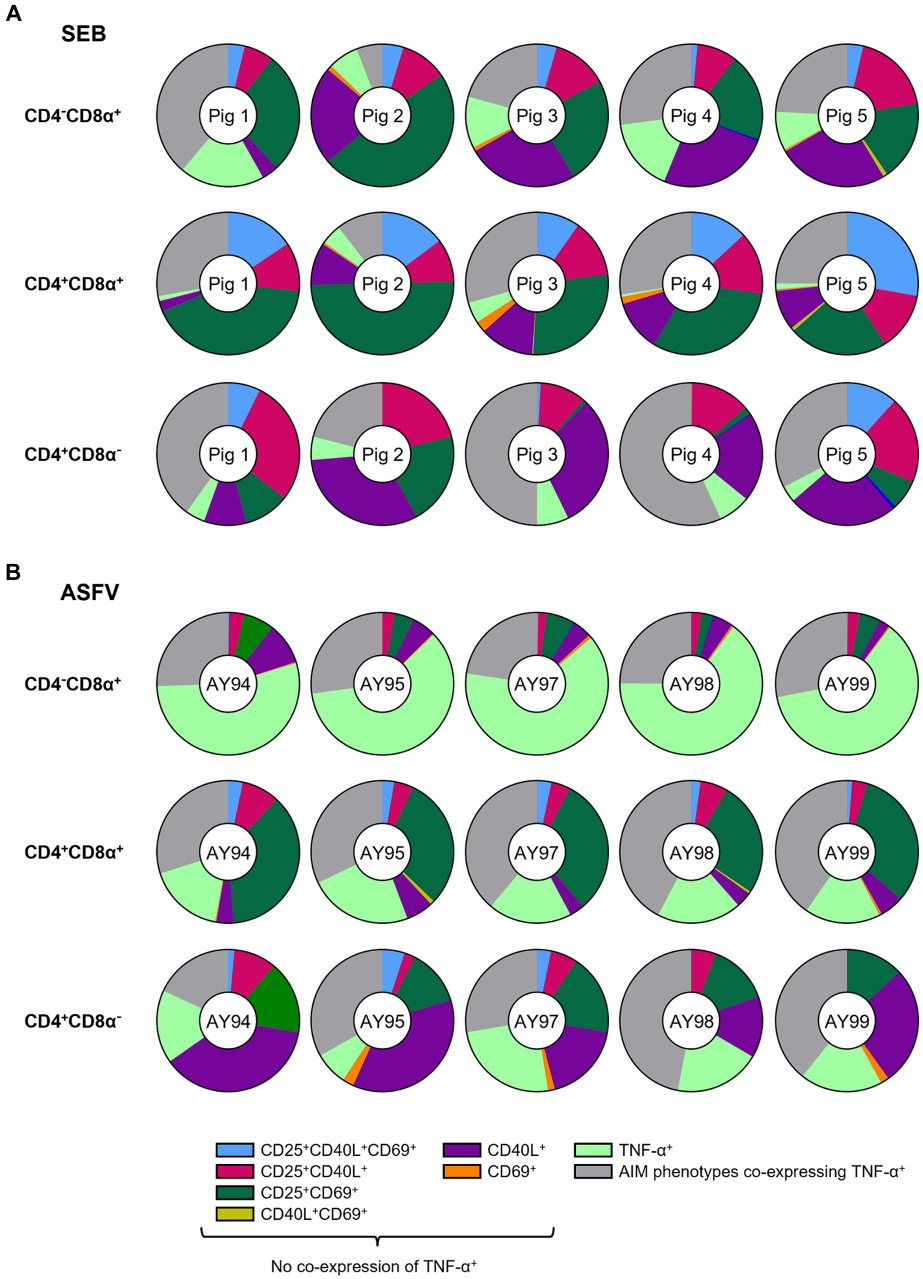
Relative distribution of AIM phenotypes within CD4/CD8α-defined T cell subsets in SEB-stimulated vs. ASFV-stimulated PBMC cultures. **(A)** Doughnut charts of AIM phenotypes in SEB-stimulated samples generated by Boolean gating in CD4^−^CD8α^+^ (top row), CD4^+^CD8α^+^ (middle row) and CD4^+^CD8α^−^ (bottom row) T cells. Each doughnut represents the PBMC sample of one pig. Different phenotypes are indicated by different colors with all AIM phenotypes co-expressing TNF-α summarized in gray. CD25^−^CD40L^−^CD69^−^TNF-α^−^ and CD25 single^+^ T cell phenotypes are not shown. **(B)** Doughnut charts of AIM phenotypes in ASFV Estonia 2014-stimulated samples generated by Boolean gating in CD4^−^CD8α^+^ (top row), CD4^+^CD8α^+^ (middle row) and CD4^+^CD8α^−^ (bottom row) T cells. Each doughnut represents the PBMC sample of one pig. Different phenotypes are indicated by different colors with all AIM phenotypes co-expressing TNF-α summarized in gray. CD25^−^CD40L^−^CD69^−^TNF-α^−^ and CD25 single^+^ T cell phenotypes are not shown.

Omitting CD25 single^+^ T cells, CD25^+^CD69^+^ (dark green), CD25^+^CD40L^+^ (pink) and CD40L single^+^ (purple) constituted the most frequent AIM^+^ T cell phenotypes after SEB stimulation (Figure 4A) with CD25^+^CD69^+^ in CD4^+^CD8α^+^ T cells reaching an average of 34.7% when AIM^neg^TNF-α^neg^ and CD25 single^+^ T cells were excluded from the calculation (referred to as “ATC excluded” [from **A**IM^neg^**T**NF-α^neg^ **C**D25 single^+^] from now on; percentages in total T cells given in Supplementary Table 1). While CD25^+^CD40L^+^ (pink, average 11.5% in CD4^−^CD8α^+^, 12.3% in CD4^+^CD8α^+^, 18.6% in CD4^−^CD8α^+^ of ATC-excluded) and CD40L single^+^ (purple, average 20.3% in CD4^−^CD8α^+^, 9% in CD4^+^CD8α^+^, 23.3% in CD4^−^CD8α^+^ of ATC-excluded) AIM phenotypes were frequent in all T cell subsets, CD25^+^CD40L^+^CD69^+^ (light blue, average 3.6% in CD4^−^CD8α^+^, 16.2% in CD4^+^CD8α^+^, 4% in CD4^−^CD8α^+^ of ATC-excluded) were mostly found within CD4^+^CD8α^+^ T cells.

Among ASFV Estonia-stimulated samples, TNF-α single^+^ (light green) and CD25^+^CD69^+^ (dark green) T cells were the most prominent phenotypes (Figure 4B). In contrast to SEB, however, differences between CD4/CD8α-defined T cell subsets were more pronounced. TNF-α single^+^ (light green) vastly dominated within CD4^−^CD8α^+^ T cells with an average of 60.9% within ATC-excluded. While CD25^+^CD69^+^ T cells (dark green) were frequent within both CD4^+^CD8α^+^ (31.2% of ATC-excluded) and CD4^+^CD8α^−^ T cells (15.1% of ATC-excluded), CD40L single^+^ were largely confined to the CD4^+^CD8α^−^ T cell subset (purple, average 25.9% of ATC-excluded). Overall, CD25^+^CD69^+^ and CD40L single^+^ were the most prominent AIM^+^ phenotypes elicited by SEB and ASFV Estonia stimulation.

Of note, frequencies of AIM^+^ T cell phenotypes co-expressing TNF-α (gray) varied between T cell subsets and stimulations (SEB: 23.4% in CD4^−^CD8α^+^, 24% in CD4^+^CD8α^+^, 40.1% in CD4^−^CD8α^+^ of ATC-excluded; ASFV: 25.7% in CD4^−^CD8α^+^, 36.7% in CD4^+^CD8α^+^, 34% in CD4^−^CD8α^+^of ATC-excluded). Focusing in more detail on AIM^+^ TNF-α^+^ T cell phenotypes revealed further differences between SEB and ASFV Estonia-stimulated PBMC cultures (Supplementary Figures 5A,B). While AIM^+^ TNF-α^+^ T cell phenotypes in SEB-stimulated samples were dominated by CD40L^+^TNF-α^+^ (rust-red) and CD25^+^CD40L^+^TNF-α^+^ (light blue) T cells, CD25^+^TNF-α^+^ T cells (green) were the most prominent phenotype in ASFV Estonia samples. CD25^+^CD40L^+^CD69^+^TNF-α^+^ T cells (maroon) were mostly restricted to CD4^+^ T cells, being present in CD4^+^CD8α^+^ and CD4^+^CD8α^−^ subsets after both stimulations. Therefore, TNF-α production in SEB-stimulated cells seems to be largely associated with CD40L expression, whereas it is rather affiliated with CD25 in ASFV Estonia-stimulated samples. In accordance with this, when analyzing ICOS^+^ T cell phenotypes in combination with the other AIMs and TNF-α in ASFV Estonia-samples, ICOS was preferentially co-expressed with CD25 (Supplementary Figure 6).

### 3.5 Multiple AIM^+^ phenotypes expand in response to ASFV

**Figure 5:**
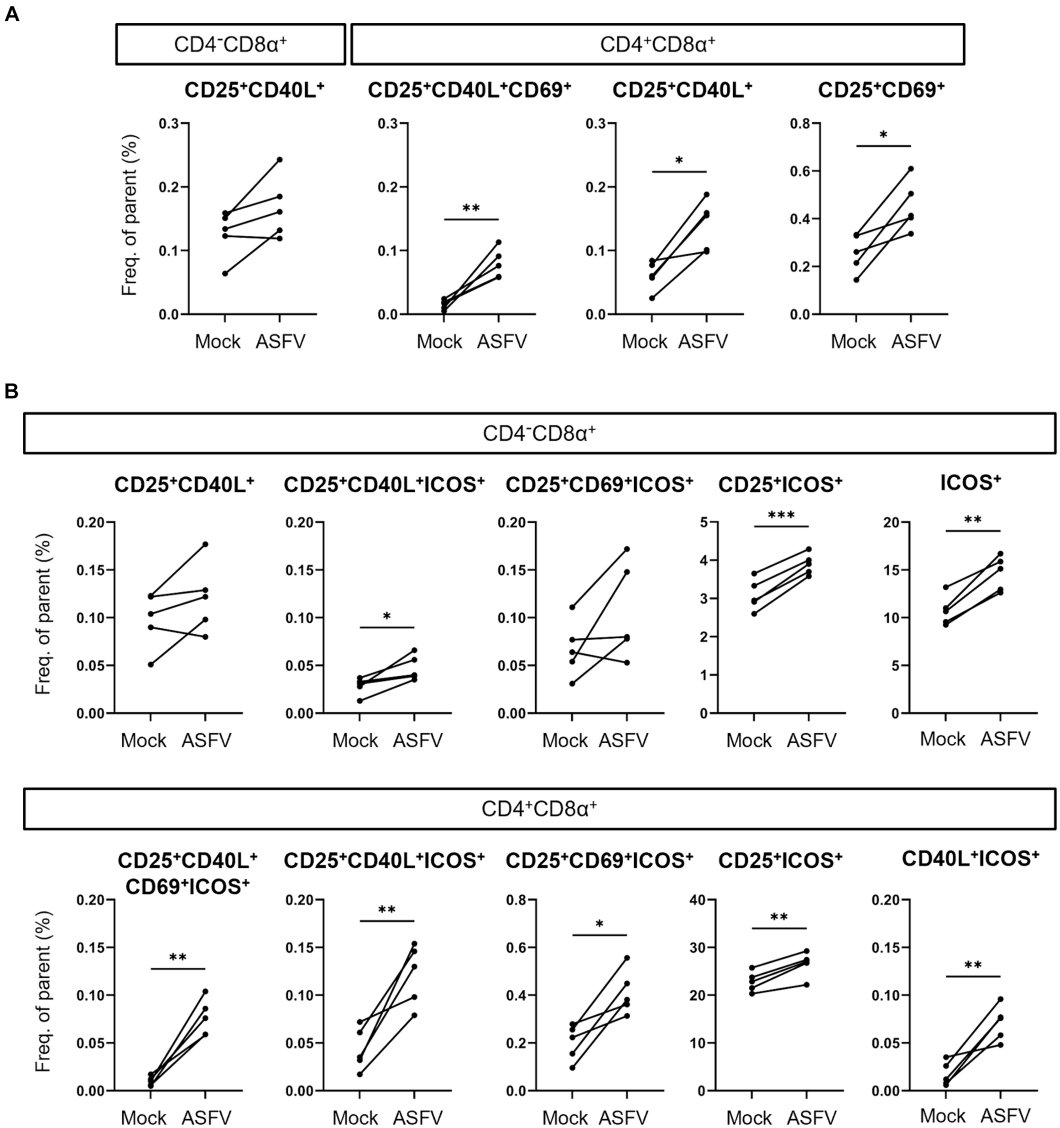
Induction of AIM^+^ CD4/CD8α-defined T cell phenotypes after ASFV Estonia 2014 stimulation. **(A,B)** Frequencies of selected AIM^+^ phenotypes within CD4^−^CD8α^+^ and CD4^+^CD8α^+^ T cells in mock-inoculated (Mock) vs. ASFV Estonia 2014 (ASFV)-stimulated samples. Boolean gating of AIMs was performed excluding **(A)** or including analysis of ICOS expression **(B)**. Each dot represents data from one animal (*n* = 5). Asterisks indicate significant differences between groups (^*^p ≤ 0.05, ^**^p ≤ 0.01, ^***^p ≤ 0.001).

Finally, we dissected which combinations of AIMs expanded in response to ASFV stimulation and therefore prove valuable for the identification of antigen-specific T cells. Consequently, we applied further Boolean gating of CD25^+^, CD40L^+^ and CD69^+^ within CD4/CD8α-defined T cell subsets, omitting analysis of TNF-α expression to focus exclusively on expression of AIMs. Significant increases in frequencies were observed for CD25^+^CD40L^+^CD69^+^, CD25^+^CD40L^+^ and CD25^+^CD69^+^ phenotypes in CD4^+^CD8α^+^ T cells (Figure 5A). For CD4^+^CD8α^−^ T cells, consisting mainly of naïve CD4 T cells in pigs (35), no significant differences between mock and ASFV-treated cultures were found (data not shown). When including ICOS in the AIM panel, CD25^+^CD40L^+^ICOS^+^, CD25^+^ICOS^+^ and ICOS^+^ phenotypes were significantly upregulated in the CD4^−^CD8α^+^ T cell subset in ASFV-cultures (Figure 5B, top). Within CD4^+^CD8α^+^ T cells, five AIM^+^ phenotypes co-expressing ICOS showed significant expansion following ASFV restimulation: CD25^+^CD40L^+^CD69^+^ICOS^+^, CD25^+^CD40L^+^ICOS^+^, CD25^+^CD69^+^ICOS^+^, CD25^+^ICOS^+^ and CD40L^+^ICOS^+^ (Figure 5B, bottom). This suggests that the addition of ICOS improves the breadth of the investigated AIM panel. In summary, multiple AIM^+^ phenotypes expanded in ASFV-primed cells after ASFV restimulation, most notably within CD4^+^CD8α^+^ T cells.

## 4 Discussion

AIMs have become a widely used tool to identify the full breadth of antigen-specific CD4 and CD8 T cells following short-term *in vitro* restimulation (8). However, in livestock species, their use has been very limited, probably due to a perceived lack of antibodies addressing AIMs established for human T cells. Here, we made use of species specific or cross-reactive antibodies for pigs, binding to CD25, CD69, CD40L and ICOS to investigate activated T cells following polyclonal, oligoclonal or antigen (re-)stimulation.

PMA/ionomycin stimulation resulted in a substantial increase of T cells expressing CD25, CD69 and CD40L, suggesting that all three molecules are suitable for the identification of activated T cells. Previously, we reported similar results for ICOS following ConA stimulation (13). However, stimulation by either PMA/ionomycin or ConA is very potent in T cells and therefore does not allow immediate conclusions on the suitability of CD25, CD69, CD40L or ICOS to identify antigen-specific T cells. As an intermediate to antigen recall, we initially tested our AIM panel on SEB stimulated PBMCs. SEB is a group II superantigen, binding to the α-chain of MHC class II molecules and the Vβ chain of the TCR (40). In human PBMCs, stimulation with SEB (1 μg/mL) results in up to 10% TNF-α producing (41) and 30% CD40L expressing CD4 T cells (9). Although we and others worked with similar concentrations for porcine PBMC [500 ng/mL to 1 ug/mL (11, 42)], percentages of activated phenotypes in porcine T cells are lower, e.g., in total CD4 T cells approx. 1% of cells become CD40L^+^ (11). In our study 5%–14% and 1.7%–5% within CD4^+^CD8α^+^ T cells expressed CD40L or TNF-α, respectively (Figure 2B). This suggests that SEB could have a lower affinity for porcine MHC-II α-chains or TCR-Vβ similar to what has been reported for mice (43). Although this could be seen as a disadvantage on the overall suitability of SEB to stimulate porcine T cells, it might be beneficial for the overall goal: to use AIMs to identify memory T cells capable of recall responses, where affinity between MHC/peptide and the TCR is also rather low. Indeed, our results show that CD25^+^CD69^+^ is a prominent phenotype induced by both SEB and ASFV (re-)stimulation, at least in CD4^+^CD8α^+^ T cells.

At the beginning of our work on AIMs for porcine T cells, we also tested antibodies against CD71, CD137 and CD274. Although CD71 is less frequently analyzed in human T cells (8), its suitability as an AIM for T cells from mice and humans has been suggested (44) and a cross-reactive antibody (clone T56/14) for pig was reported (45). However, in our hands, the antibody did not show any binding on resting or ConA-stimulated T cells (data not shown). Similarly, cross-reactive antibodies for human AIMs CD137 (clone 4B4-1) and CD274 (PD-L1, clone 29E.2A3) have been mentioned (46), but again both antibodies did not bind to T cells or other cells in porcine PBMCs in our hands. A pig specific mAb for porcine CD137 has been reported (47) but to our knowledge is currently not commercially available and was not investigated in this study. Collectively, this illustrates that the toolbox for AIMs in pigs is still limited but our work shows that addressing CD25, CD69, CD40L and ICOS in combination should allow the identification of activated CD4 and CD8 T cells in different experimental settings.

Different to cytokines, which for FCM analysis require intracellular staining, AIMs are membrane bound molecules. This allows for cell sorting and further downstream analyses, for example *in vitro* testing or bulk transcriptome analysis. However, as in previous studies on equine and porcine T cells (11, 48), CD40L expression was investigated after fixation and permeabilization of cells. This is due to the extremely short half-life of CD40L on the cell membrane (49), limiting possibilities to identify it while on the cell surface. To overcome the need of permeabilization for sensitive CD40L detection, two strategies have been developed in the past. One is to place CD40L mAbs into the culture during the period of *in vitro* stimulation. This requires addition of monensin to the culture, to prevent degradation of the fluorochromes which partially become internalized together with their antibodies during the cultivation period (19). This approach was also successfully used for porcine CD4 T cells, allowing the combined analysis of CD40L in combination with either TNF-α or IFN-γ (50). However, when we tried this in combination with the analysis of CD25 and CD69, the expression of these molecules was severely impaired by the monensin (data not shown). An alternative to this is the addition of CD40 blocking antibodies (9, 20), a standard procedure in human AIM assays when CD40L is included. We have not tested this approach in our experiments, but several putatively pig cross-reactive antibodies (rabbit polyclonal PA5-27419; rabbit polyclonal PA1-31075; mouse anti-human, clone 647CT13.2.4; mouse anti-human, clone LOB7/6; mouse anti-human, clone 9G10) were already unsuccessfully tested by Ebner and colleagues (F. Ebner, personal communication). Hence, a sorting of antigen-specific porcine T cells could either be performed based on CD25/CD69/ICOS co-expression (or subsets thereof, our findings) or on CD40L only (50). Nevertheless, further testing of antibodies for blocking porcine CD40 would strengthen AIM guided sorting of re-activated memory T cells.

In our experiments we compared AIM expressing T cells against the capacity for TNF-α production. TNF-α was chosen based on previous work, showing that frequencies of TNF-α and IFN-γ producing CD4 and CD8 T cells are similar following PMA/ionomycin stimulation. However, TNF-α production was also found in CD4^+^CD8α^−^ T cells, while IFN-γ production was largely confined to CD4^+^CD8α^+^ and CD4^−^CD8α^+^ T cells (24). In addition, IFN-γ and TNF-α are often co-produced, both after PMA/ionomycin stimulation (24) and for example in influenza or porcine reproductive and respiratory syndrome virus (PRRSV) recall assays (51–54). Nevertheless, all those publications show that IFN-γ single producing T cells are frequently present, suggesting that IFN-γ production will also correlate with the AIMs investigated in our work. This clearly warrants future investigations. Of note, TNF-α has been subject to limited investigation in the context of ASFV restimulation (55), but our data show that a considerable proportion of CD4^−^CD8α^+^ T cells can produce this cytokine in a recall assay. The CD4^−^CD8α^+^ T cells in our phenotyping panel should largely consist of conventional CD8 T cells, although a minor subset of CD2^+^ γδ T cells can also have this phenotype (56). However recall stimulation of cells from animals recovered from a low virulent isolate of ASFV did not induce secretion of IFN-γ or TNF-α from CD3^+^γδTCR1^+^ cells (55). Nonetheless, a more detailed analysis of TNF-α production in CD8 T cells in the context of different ASFV infections is of interest and should be addressed in future studies.

Finally, our data show that AIM combinations of CD25, CD40L, CD69 and ICOS identify phenotypes that expand significantly within CD4^+^CD8α^+^ T cells following ASFV restimulation. The high percentage of CD25^+^ICOS^+^ cells within CD4^+^CD8α^+^ T cells (also within mock cultures, Figure 5B, bottom) suggests that this phenotype contains a considerable proportion of Treg cells. Porcine Treg cells mainly have a CD25^high^ phenotype but also subsets of CD25^dim/intermediate^ CD4 T cells can express Foxp3 (57). This demonstrates the need to combine CD25 with other AIMs. Indeed, combinations of three or four AIMs identified much smaller subsets of CD4^+^CD8α^+^ T cells showing an increase after ASFV restimulation (Figure 5B, bottom) with frequencies being closer to what can be expected in the context of *in vitro* antigen recall. Of note, we also identified ASFV induced increases of CD25^+^ICOS^+^ and single ICOS^+^ CD4^−^CD8α^+^ T cells (Figure 5B, top), potentially arising from ICOS^low^ CD4^−^CD8α^+^ T cells (Figure 3A). Mouse splenic and blood-derived CD8 T cells have been reported to be largely ICOS^−^ (39, 58), but more recently it was shown that ICOS drives the generation of CD8 tissue resident memory T (Trm) cells (59). This indicates that further investigations on ICOS expressing CD8 T cells and its role as an AIM in pigs are justified. Together, this shows the versatility of our panel, providing avenues to investigate those CD25/CD40L/CD69/ICOS AIM phenotypes in pigs in future experiments, both in the context of infection and immunization studies.

## Data availability statement

The raw data supporting the conclusions of this article will be made available by the authors, without undue reservation.

## Ethics statement

The animal study was approved by the Animal Welfare and Ethical Review Board (AWERB) at The Pirbright Institute. The study was conducted in accordance with the local legislation and institutional requirements.

## Author contributions

MM: Writing – review & editing, Methodology, Investigation. PT: Writing – review & editing, Supervision, Project administration, Methodology, Investigation, Funding acquisition. RI: Writing – review & editing, Resources. CN: Writing – review & editing, Supervision, Project administration, Funding acquisition. WG: Writing – original draft, Supervision, Project administration, Funding acquisition, Conceptualization. SS: Writing – original draft, Visualization, Supervision, Project administration, Funding acquisition, Formal analysis, Conceptualization.
