# Supplementary material for: Investigation of activation-induced markers (AIM) in porcine T cells by flow cytometry
Source: Front Vet Sci. 2024 May 29;11:1390486. doi: 10.3389/fvets.2024.1390486 (PMC11168203; doi:10.3389/fvets.2024.1390486)
Supplement: Supplementary file 2 [file Data_Sheet_1.PDF]

Supplementary Material

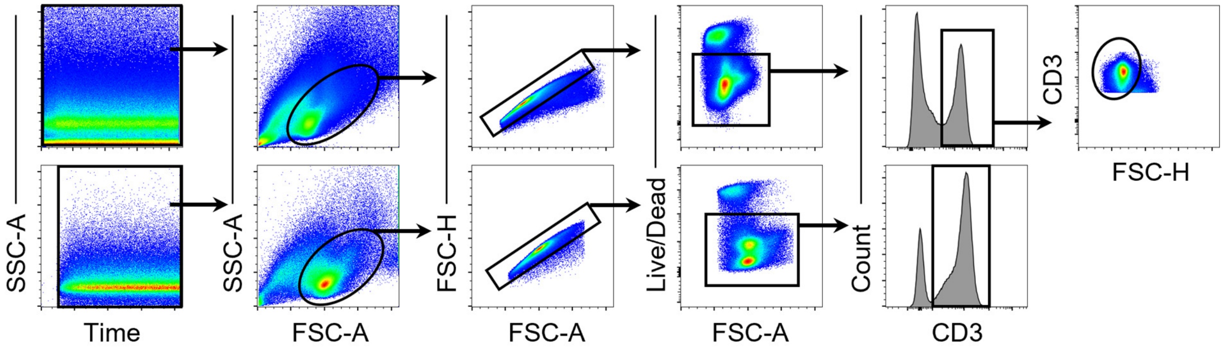

Supplementary Figure 1. FCM gating strategy of $CD3^{+}$ T cells in PBMCs. Representative gating strategy for unstimulated, PMA- and SEB-stimulated samples (top panel) as well as mock-inoculated and ASFV Estonia 2014-stimulated samples (bottom panel). Cells were gated according to their light scatter properties and sub-gated for live cells. A gate was set on $CD3^{+}$ cells and a staining artefact was gated out by removing $FSC\text{-}H^{+}$ cells.

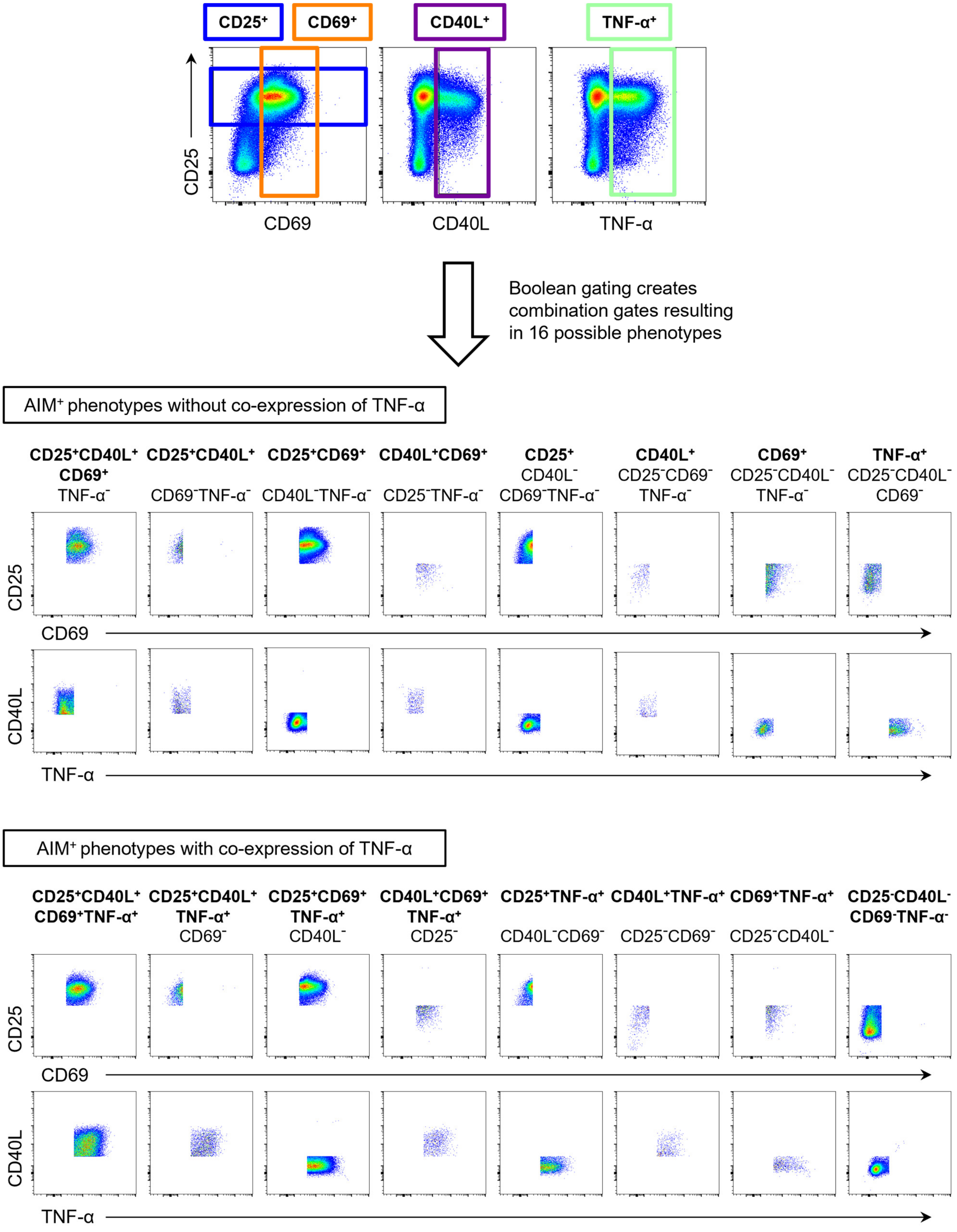
CD25^{+}
CD69^{+}
CD40L^{+}
TNF-α^{+}
CD25
CD69
CD40L
TNF-α
Boolean gating creates combination gates resulting in 16 possible phenotypes
AIM^{+} phenotypes without co-expression of TNF-α
CD25^{+}CD40L^{+}CD69^{+} TNF-α^{-}
CD25^{+}CD40L^{+} CD69^{-}TNF-α^{-}
CD25^{+}CD69^{+} CD40L^{-}TNF-α^{-}
CD40L^{+}CD69^{+} CD25^{-}TNF-α^{-}
CD25^{+} CD40L^{-} CD69^{-}TNF-α^{-}
CD40L^{+} CD25^{-}CD69^{-} TNF-α^{-}
CD69^{+} CD25^{-}CD40L^{-} TNF-α^{-}
TNF-α^{+} CD25^{-}CD40L^{-} CD69^{-}
CD25
CD69
CD40L
TNF-α
AIM^{+} phenotypes with co-expression of TNF-α
CD25^{+}CD40L^{+} CD69^{+}TNF-α^{+}
CD25^{+}CD40L^{+} TNF-α^{+} CD69^{-}
CD25^{+}CD69^{+} TNF-α^{+} CD40L^{-}
CD40L^{+}CD69^{+} TNF-α^{+} CD25^{-}
CD25^{+}TNF-α^{+} CD40L^{-}CD69^{-}
CD40L^{+}TNF-α^{+} CD25^{-}CD69^{-}
CD69^{+}TNF-α^{+} CD25^{-}CD40L^{-}
CD25^{-}CD40L^{-} CD69^{-}TNF-α^{-}
CD25
CD69
CD40L
TNF-α

Supplementary Figure 2. Boolean gating illustrated on PMA-stimulated samples. Total CD25$^+$, CD69$^+$, CD40L$^+$ and TNF-α$^+$ cells were gated within CD3$^+$ T cells. These gates were used to create Boolean 'AND' combination gates resulting in 16 possible combinations. 8 out of those 16 combinations encompass AIM$^+$ phenotypes without co-expression of TNF-α including TNF-α single$^+$ cells (top panel). The other 8 combinations encompass AIM$^+$ phenotypes with co-expression of TNF-α (bottom panel). FCM plots show expression of CD25, CD69, CD40L and TNF-α within respective phenotypes.

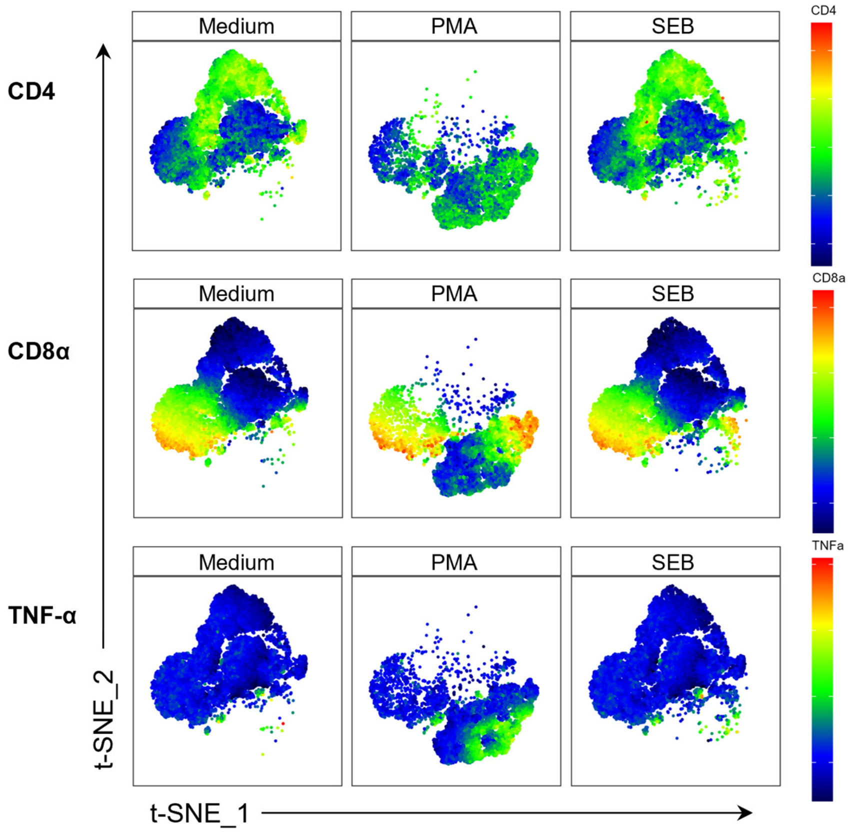

Supplementary Figure 3. Expression levels of CD4, CD8α and TNF-α in $CD3^+$ T cell clusters after stimulation with PMA/ionomycin or SEB. Live $CD3^+$ T cells from unstimulated (Medium), SEB-stimulated and PMA-stimulated cultures were clustered using the t-SNE algorithm. Relative expression levels of CD4, CD8α and TNF-α within clusters are colored from high (red) to low (blue).

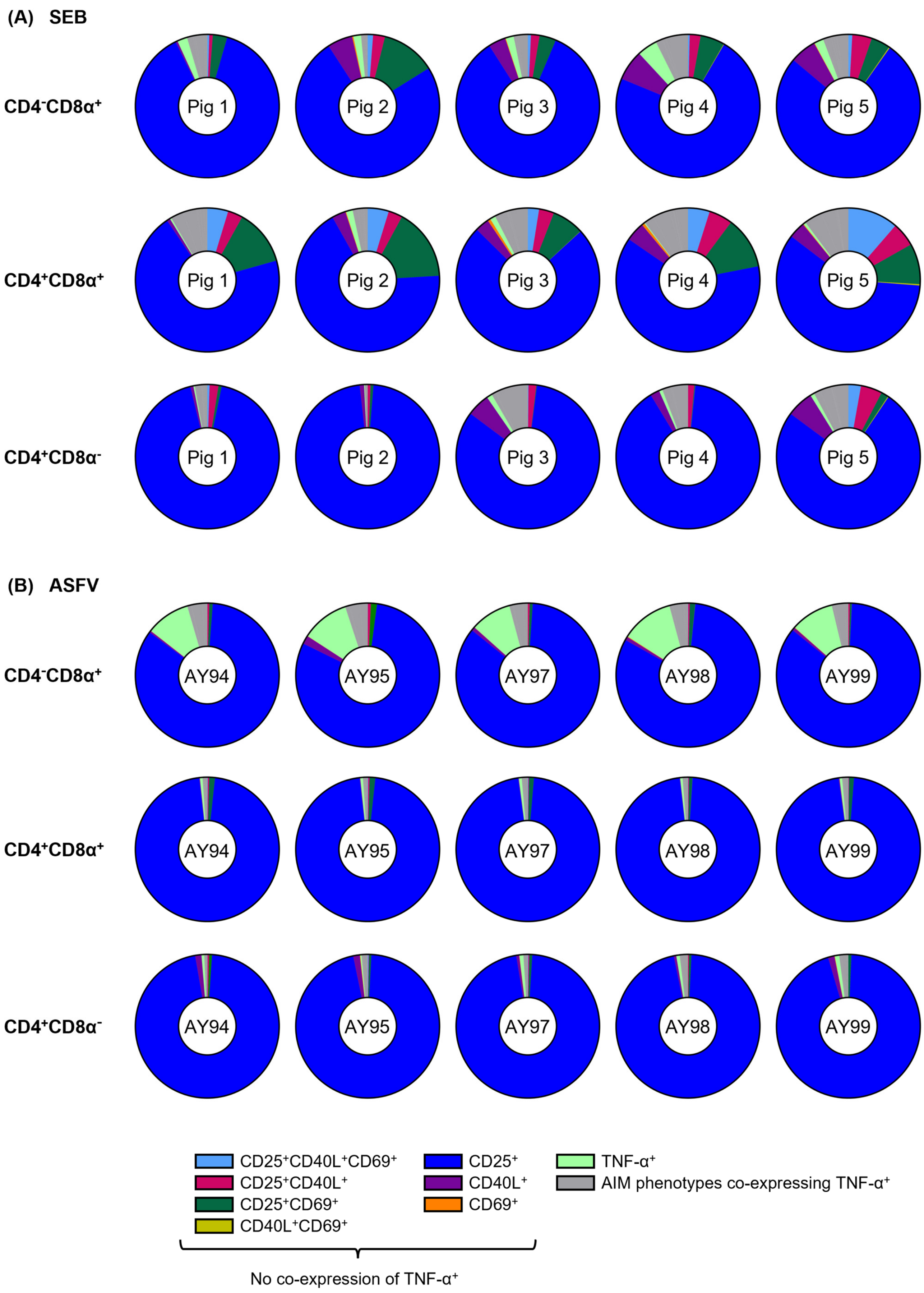
(A) SEB
CD4⁻CD8α⁺
CD4⁺CD8α⁺
CD4⁺CD8α⁻
Pig 1
Pig 2
Pig 3
Pig 4
Pig 5
(B) ASFV
AY94
AY95
AY97
AY98
AY99
CD25⁺CD40L⁺CD69⁺
CD25⁺CD40L⁺
CD25⁺CD69⁺
CD40L⁺CD69⁺
CD25⁺
CD40L⁺
CD69⁺
TNF-α⁺
AIM phenotypes co-expressing TNF-α⁺
No co-expression of TNF-α⁺

Supplementary Figure 4. Relative distribution of AIM phenotypes within CD4/CD8α-defined T cell subsets in SEB- and ASFV Estonia 2014-stimulated PBMC cultures including CD25 single^{+} T cells. **(A-B)** Doughnut charts of AIM phenotypes in SEB-stimulated **(A)** and ASFV Estonia 2014-stimulated samples **(B)** generated by Boolean gating in CD4^{-}CD8α^{+} (top row), CD4^{+}CD8α^{+} (middle row) and CD4^{+}CD8α^{-} (bottom row) T cells. Each doughnut represents the PBMC sample of one pig. Different phenotypes are indicated by different colors with all AIM phenotypes co-expressing TNF-α summarized in grey. CD25^{-}CD40L^{-}CD69^{-}TNF-α^{-} T cells are not shown.

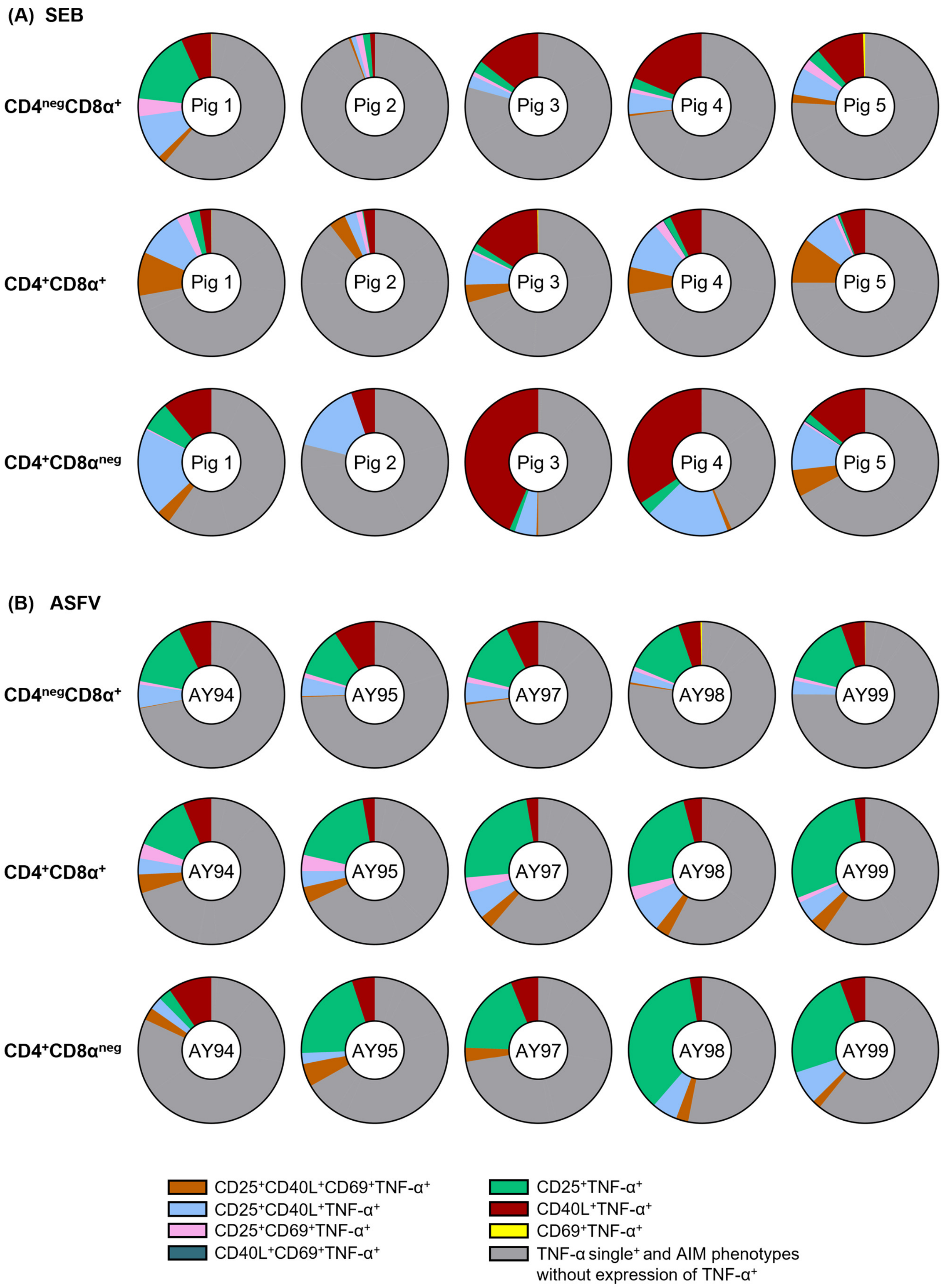
(A) SEB
CD4negCD8α+
CD4+CD8α+
CD4+CD8αneg
Pig 1
Pig 2
Pig 3
Pig 4
Pig 5
(B) ASFV
CD4negCD8α+
CD4+CD8α+
CD4+CD8αneg
AY94
AY95
AY97
AY98
AY99
CD25+CD40L+CD69+TNF-α+
CD25+CD40L+TNF-α+
CD25+CD69+TNF-α+
CD40L+CD69+TNF-α+
CD25+TNF-α+
CD40L+TNF-α+
CD69+TNF-α+
TNF-α single+ and AIM phenotypes without expression of TNF-α+

Supplementary Figure 5. Relative distribution of AIM^+ TNF-α^+ phenotypes within CD4/CD8α-defined T cell subsets in SEB- and ASFV Estonia 2014-stimulated PBMC cultures **(A-B)** Doughnut charts of AIM phenotypes in SEB-stimulated **(A)** and ASFV Estonia 2014-stimulated samples **(B)** generated by Boolean gating in $CD4^-CD8\alpha^+$ (top row), $CD4^+CD8\alpha^+$ (middle row) and $CD4^+CD8\alpha^-$ (bottom row) T cells. Each doughnut represents the PBMC sample of one pig. AIM^+ TNF-α^+ phenotypes are highlighted by different colors with all AIM phenotypes without TNF-α expression including TNF-α $single^+$ T cells summarized in grey. $CD25^-CD40L^-CD69^-$TNF-α^- T cells are not shown.

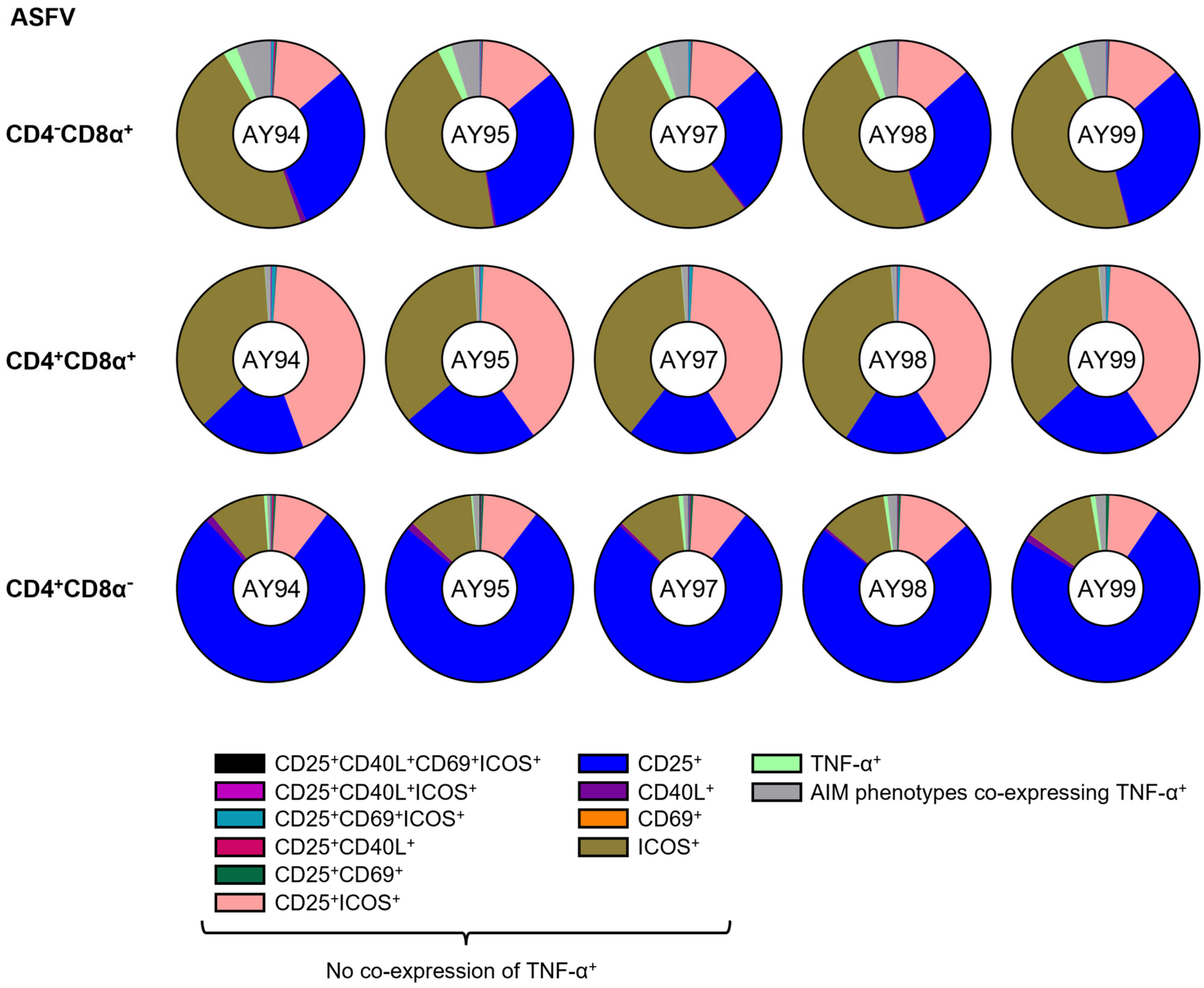

Supplementary Figure 6. Relative distribution of AIM phenotypes within CD4/CD8α-defined T cell subsets in ASFV Estonia 2014-stimulated PBMC cultures including CD25 single^{+} and ICOS^{+} T cell phenotypes. Doughnut charts of AIM phenotypes in ASFV Estonia 2014-stimulated samples generated by Boolean gating in CD4^{-}CD8α^{+} (top row), CD4^{+}CD8α^{+} (middle row) and CD4^{+}CD8α^{-} (bottom row) T cells. Each doughnut represents the PBMC sample of one pig. Different phenotypes are indicated by different colors with all AIM phenotypes co-expressing TNF-α summarized in grey. CD25^{-}CD40L^{-}CD69^{-}ICOS^{-}TNF-α^{-} T cells are not shown.
